# A huge left Staghorn kidney, a case report of inevitable open surgery: a case report

**DOI:** 10.4076/1757-1626-2-8234

**Published:** 2009-09-11

**Authors:** Mohammad Kazem Moslemi, Ali Safari

**Affiliations:** 1 Urology Department, Kamkar Hospital, Qom Medical Sciences University, School of MedicineQomIran; 2 Urology Department, Kamkar Hospital, AZAD Medical Sciences University, School of MedicineQomIran

## Abstract

**Introduction:**

Urolithiasis is a very common problem, especially in industrialized societies. Extracorporeal shockwave lithotripsy, percutaneous nephrolithotomy, and transureteral lithotripsy are effective less invasive treatments of renal and ureteral stones. Open stone surgery is used less commonly due to its invasiveness and availability of above mentioned techniques. We introduce a case, that due to heavy and complex stone burden and increased chance of failure of percutaneous nephrolithotomy, Open stone surgery is performed for stone removal.

**Case presentation:**

The patient is a 55-years-old Iranian patient that referred to urology department due to a large left Staghorn kidney. After full evaluation and due to extensive spread of stone horns to the even peripheral calyces, open stone surgery performed successfully, that postoperative dynamic renal studies revealed, near normal functional left kidney.

**Conclusion:**

In spite of wonderful advances in endourologic stone surgery, open stone surgery still has its role, but it must be done in experienced centers with good surgical expertise to retain good and acceptable functional kidney, postoperatively.

## Case presentation

The patient is a 55-years fatty old Iranian male, with BMI of 30, that complained from occasional left flank pain, from 1 to 2 years before admission. He was also complaining of irritative lower urinary tract symptoms; dysuria, frequency in moderate severity. 1 to 2 bouts of upper UTI also noted in his past history. No history of other medical or surgical problems was noted.

In renal ultrasonography, a large left staghorn kidney was reported.

**Figure 1.**
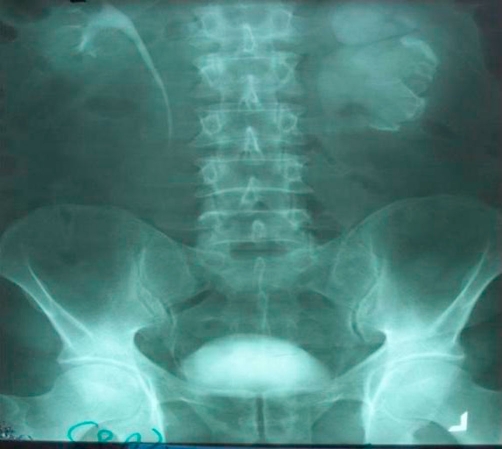
IVP, just before surgery, large staghorn of left kidney is evident.

In IVP a huge semiopaque density is seen in left kidney (Figure 1). The urine culture was positive for E coli growth.

Due to large volume of stone and its complexity, the patient is scheduled for left anatrophic nephrolithotomy. He was not candidate for PCNL, It was impractical due to volume of the stone and increased rate of complications and failure.

**Figure 2.**
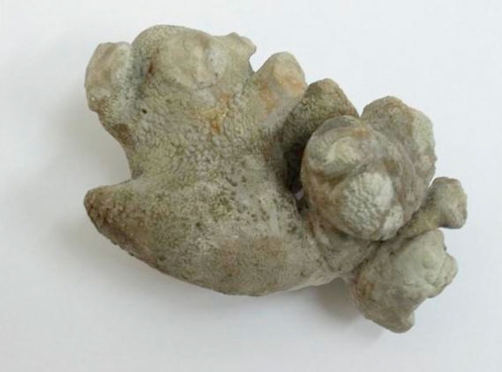
The stone that removed completely, its weight was 123 grams.

The patient underwent open stone surgery, with left flank incision, after ligating the renal artery and opening the kidney with nephrotomy incision, the stone removed completely (Figure 2) within 5 min after artery ligation. The ligature released after 9 min.

The stone weight was 123 grams. Stone biochemical analysis was in favor of struvite stone.

**Figure 3.**
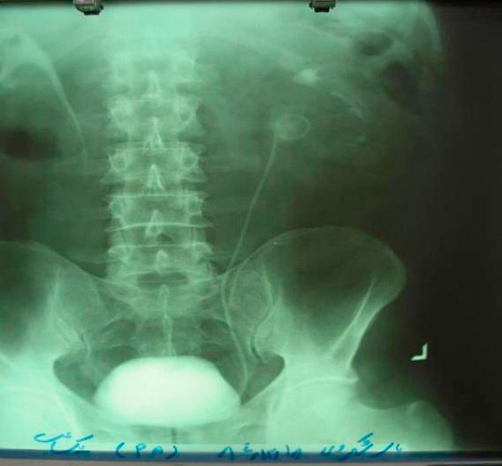
IVP, two month after surgery, showing normal secretion of kidneys.

In IVP that was done 2 month after surgery, normal function of the kidneys, especially, operated left one, was noted (Figure 3). Urine cultures, one month, two month, 6 month and, one and two years after operation, all were negative. Also ultrasonography of the kidneys 6 month, one year and two years after operation were unremarkable.

## Discussion

ESWL, PCNL and TUL are advanced noninvasive or less invasive techniques of renal and ureteral stones treatment. In the last few decades, with the improvement in endourological surgery and the invention and evolution of extracorporeal shock-wave lithotripsy, the indications for open surgery in stone disease have become rare, although open surgery still has a role in selected cases. Open stone surgery, are losing its role day and day, but sometimes its using becomes inevitable due to patients characteristics, failure of primary therapy for stone removal [1], complex stone burden, renal anatomic problems (such as ureteropelvic junction obstruction and infundibular stenosis with or without renal caliceal diverticulum) or an additional target of therapy apart from stone removal such as the treatment of stones in a primary obstructive mega ureter [2]. The level of evidence for the currently available guidelines is not adequate, mainly because of lack of properly designed, large prospective randomized trials that compare different options [3]. Open surgery if required, may be replaced by laparoscopic procedures. Nevertheless, centers with the equipment, expertise and experience in the surgical treatment of renal stones report a need for open surgery in 1-5 4% of cases [1].

We have some types of open stone surgery, like pyelolithotomy that is used in the case of renal pelvic stones or renal pelvic stones with extension to one or two major calyces (that extended pyelolithotomy is used), simple nephrolithotomy or anatrophic nephrolithotomy (that is used in the case of simple calyceal stones or Staghorn stones, respectively).

PCNL is a valuable treatment option for complete Staghorn stones with a stone-free rate approaching that of open surgery. Moreover, it has the advantages of lower morbidity, shorter operative time, shorter hospital stay and earlier return to work [4]. Percutaneous nephrolithotomy (PNL) is superior to shockwave lithotripsy (SWL) or open surgery in the treatment of Staghorn calculi [5]. In a retrospective study on the 780 procedures performed for stone removal, 42 were open surgical procedures (5.4%) including pyelolithotomy and anatrophic nephrolithotomy in 29 cases (69%). The most common indications for open surgery were complex stone burden (55%); failure of extracorporeal shock wave lithotripsy or endourological treatment (29%) and anatomic abnormalities such as ureteropelvic junction obstruction, infundibular stenosis and/or renal caliceal diverticulum (24%). Average hospital stay was 6.4 days. The stone -free rate after surgery was 93%. In conclusion, open stone surgery continues to represent a reasonable alternative for a small segment of the urinary stone population [6]. Open surgery for stones of the upper urinary tract has very few indications; failure or complications of other techniques, greater than 2 cm stones, hard stones, anatomical abnormalities and complex stones. Open surgery for stone may be difficult and need specific tools. For the kidney, the anatrophic nephrotomy is an effective procedure which spares renal function [7].

## Abbreviations

ESWL: extracorporeal shockwave lithotripsy
IVP: intravenous pyelography Staghorn
OSS: open stone surgery
PCNL: percutaneous nephrolithotomy
TUL: transureteral lithotripsy
